# Depressive symptoms decrease health-related quality of life of patients with coronary artery disease and diabetes: a 12-month follow up study in primary care

**DOI:** 10.1080/02813432.2023.2233995

**Published:** 2023-07-16

**Authors:** Nina Tusa, Hannu Kautiainen, Pia Elfving, Sanna Sinikallio, Pekka Mäntyselkä

**Affiliations:** a Wellbeing services county of North Savo, Educational services, Finland; b Institute of Public Health and Clinical Nutrition, General Practice, University of Eastern Finland, Kuopio, Finland; c Siilinjärvi Health Center, Siilinjärvi, Finland; d Folkhälsan Research Center, Helsinki, Finland; e Department of Medicine, Kuopio University Hospital, Finland; f Institute of Clinical Medicine, University of Eastern Finland, Kuopio, Finland; g Terveystalo Health Services, Kuopio, Finland; h Clinical Research and Trials Centre, Kuopio University Hospital, Kuopio, Finland

**Keywords:** Primary health care, chronic diseases, hypertension, coronary artery disease, diabetes, depressive symptoms, health-related quality of life, aging population

## Abstract

**Objective:**

Health-related quality of life (HRQoL) is a multidimensional patient-related outcome. Less is known about the role of depressive symptoms on HRQoL in chronic diseases. This follow-up study analyzed depressive symptoms’ association with HRQoL change measured with 15D in patients with chronic diseases.

**Design and setting:**

A total of 587 patients from the Siilinjärvi Health Center, Finland were followed up due to the treatment of hypertension (HA), coronary artery disease (CAD) or diabetes (DM). Depressive symptoms were based on Beck Depression Inventory (BDI) (BDI ≥10 =depressive symptoms). HRQoL was assessed at the baseline and after 12 months.

**Results:**

There were 244 patients with HA (mean age 70 years, 59% women); 103 patients (72 years, 38%) with CAD and 240 with DM (67 years, 52%). The change from baseline to the 12-month follow-up in 15D was significantly different between patients without and with depressive symptoms in CAD (*p* < 0.001) and DM (*p* = 0.024). In CAD with depressive symptoms, the change was −0.064 (95% CI: −0.094 to −0.035) and in DM −0.018 (95% CI: −0.037 to 0.001). In the 15 HRQoL dimensions of 15D, a depressive symptoms-related decrease was found in three dimensions with HA, in 9 with CAD and in 7 with DM. As a function of the BDI at baseline, the 15D score decreased significantly among patients with CAD and DM.

**Conclusions:**

Depressive symptoms impact negatively on future HRQoL among primary care patients with coronary artery disease and diabetes emphasizing that mood should be acknowledged in their care and follow-up.

**Trial registration:**

Clinical Trials registration number: NCT02992431, registered December 14^th^ 2016

## Key points of the article

Health-related quality of life (HRQoL) is an important dimension of the quality and effectiveness of health care and an important predictor of mortality and morbidity.

The main finding was that baseline depressive symptoms were associated with a decrease in the health-related quality of life after 12 months of follow-up, particularly in patients with coronary artery disease and diabetes.

Beginning from the lowest scores, the severity of baseline depressive symptoms had a significant relationship with the level of deterioration in HRQoL among patients with coronary artery disease and diabetes.

A significant decrease in HRQoL related to depressive symptoms was found in various different dimensions of HRQoL.

## Introduction

Healthcare professionals strive not only to extend or save their patients’ lives but also to relieve their symptoms and improve their function and ability to participate in their daily life activities. At best, we can help patients to prevent diseases. In other words, our aim is to improve the health-related quality of life (HRQoL) of our patients.

Health-related quality of life is an important indicator that gives us the patient’s perspectives of health outcomes and the disease experience which is different from our clinical measurements. In healthcare-centred thinking, clinical measurements may appear more objective but are less meaningful to individual patients. Using HRQoL, we gain insight into any change in the physical, mental and social quality of life caused by the patient’s illness, its symptoms, worries and treatment. HRQoL is also an important dimension of the quality and effectiveness of our health care and an important predictor of mortality and morbidity [1–4]. Measures assessing HRQoL are potentially relevant patient-reported outcome measures (PROMs), which have become more important in clinical practice and trials [5].

In a large Finnish population-based study, HRQoL was measured in 29 particular diseases [6]. The common chronic diseases treated in Finnish primary health care are HA, CAD and DM. The lowest quality of life among these diseases was in DM and the highest in HA [6]. As the onset of type 2 DM and HA may be slow and very mild in terms of symptoms or even asymptomatic, the decrease of HRQoL is usually very mild at the beginning of the disease [7–10]. For example, CAD can develop slowly, but its beginning may also be dramatic and lead to acute life-saving actions. This can lead to quick changes in quality of life [11].

Depressive symptoms even without clinical depression are associated with an unhealthy lifestyle in HA patients with metabolic syndrome [12] and with mortality among patients with CAD [13] or DM [14]. Previously in Finland, patients with clinical depression have been found to have markedly lower HRQoL compared with the general population [15]. Regardless of clinical depression, depressive symptoms seem to be strongly associated with disease-specific quality of life [16].

Based on the existing scientific evidence, we can hypothesize that even milder depressive symptoms without clinical depression may contribute to decreasing HRQoL in patients with long-term chronic diseases. However, we do not know much about the relationship between depressive symptoms and HRQoL in primary care patients with common chronic diseases. Therefore, our aim in the present study was to investigate the predicting role of baseline depressive symptoms in the change of HRQoL after a 12-month follow-up in the three disease groups: hypertension, coronary artery disease and diabetes.

## Materials and methods

### Context

The data of the present study was based on a pragmatic randomized control study Participatory Patient Care Planning in Primary Care (4PHC) (ClinicalTrials.gov Identifier: NCT02992431) integrated into the practice of a health center in Siilinjärvi municipality in Finland.

### Patients

The study population consisted of adult residents (age ≥18 years) who were living in the municipality of Siilinjärvi and had hypertension (HA), coronary artery disease (CAD) or diabetes (DM). The patients were registered in the electronic patient records in the Siilinjärvi Health Center which provided the public primary health care services in the municipality. The patients were recruited from patients who had a follow-up visit due to their disease between February 2017 and March 2018. The disease groups were organized according to the degree of severity of the disease so that the HA patients had only HA, CAD patients could have also had HA and DM patients could have had all three diseases. In addition to these diseases, patients could have had other diseases. Of the DM patients, 58 had CAD (24%) and 193 (80%) had HA. The baseline results and the first follow-up results have been presented in more detail in our previous articles [17,18]. The exclusion criteria were permanent bed patient, terminal phase of any serious condition and severe decline in cognition.

**Figure 1.**
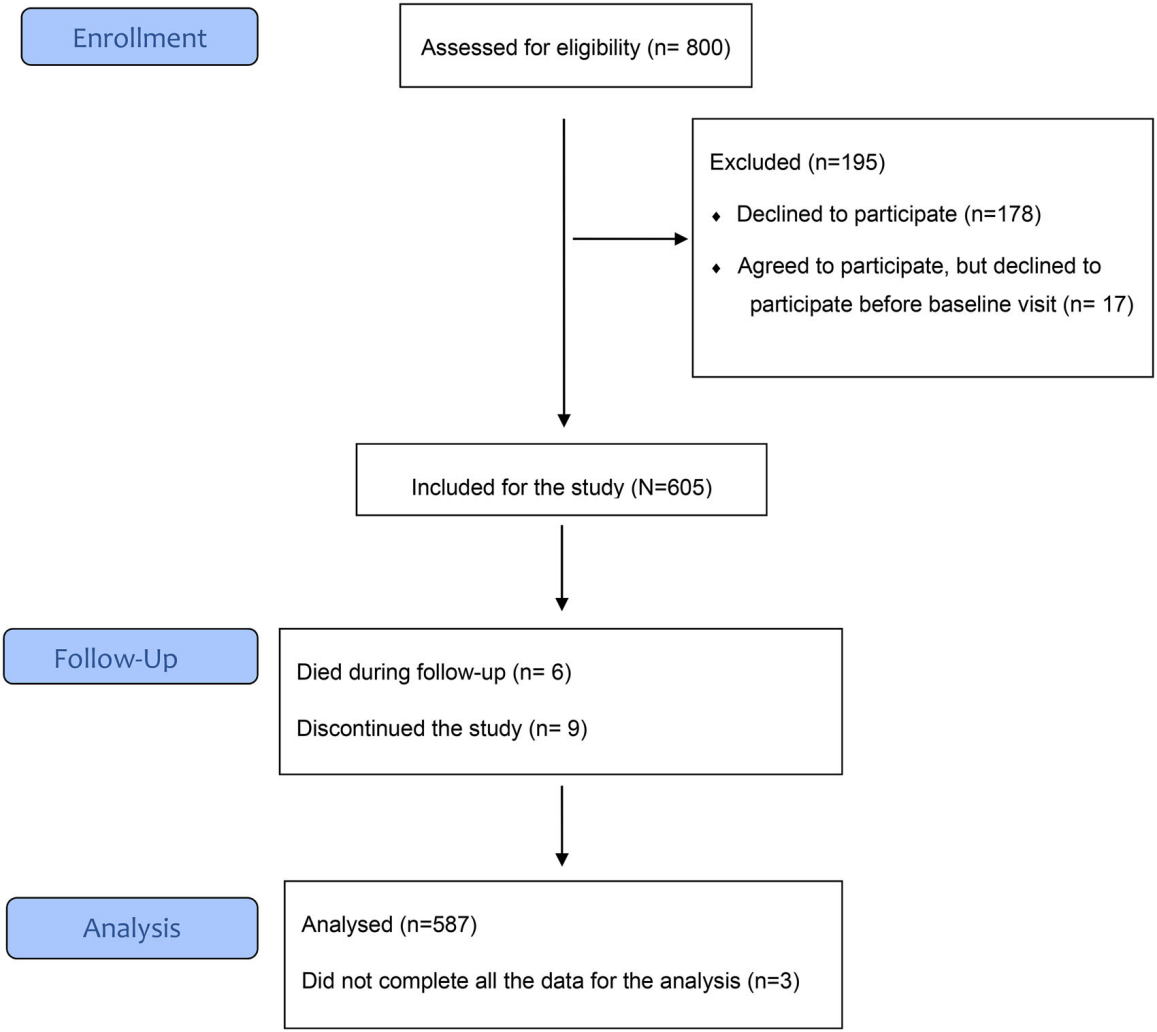
Flow diagram of the study.

Of the 800 informed patients, 622 patients agreed to participate, but 17 cancelled their participation afterwards. Of the 605 participants, 587 patients completed all the data needed for this analysis. The flow of the study is presented in Figure 1.

### Outcomes

The primary outcome was health-related quality of life measured with the 15D [19]. The 15D is a generic, comprehensive, standardized, self-administered measure of health-related quality of life. The 15D questionnaire includes the following 15 dimensions: breathing, mental function, speech (communication), vision, mobility, usual activities, vitality, hearing, eating, elimination, sleeping, distress, discomfort and symptoms, sexual activity and depression. Each dimension is divided into 5 levels. The maximum score is 1 (no problems on any dimension) and the minimum score is 0 (being dead). The smallest clinically significant change in the quality of life detected by the 15D and its dimensions is 0.015 [20]. Based on our earlier study, there were no differences between the usual care and participatory patient care plan intervention groups in HRQoL and therefore they are analysed as a whole in this study [18].

To measure the severity of depressive symptoms, participants completed the 21-item Beck Depression Inventory (BDI). Previously, in a Finnish primary care setting the study found that BDI score 10.5 was cut-off value for remission among patients with previous clinical depression [21]. In the present study, patients with a score ≥10 in the BDI were deemed to have depressive symptoms [22,23].

### Other measurements

A fasting blood sample for plasma glucose, HbA1c and low-density lipoprotein cholesterol (LDL-C) were drawn in a laboratory after 12 h of fasting. The standard procedure of the Kuopio University Hospital laboratory was used in the analysis. A trained nurse measured blood pressure in a sitting position after 10 min of sitting. Diastolic pressure (DP) and systolic pressure (SP) were recorded. The nurse also measured weight in light clothing and height, and the body mass index was calculated as weight (kg)/height (m)^2^.

The study questionnaire included questions about the educational background, relationship status, the presence of other chronic diseases, the number of drinks per week and current smoking (yes or no; the number of cigarettes per day). Physical activity was measured by the Kasari fit index [24] with a score ranging from 1 (low activity) to 100 (high activity) [25].

### Statistical methods

The descriptive statistics are presented as means with standard deviation (SD) or as counts with percentages. Group differences were evaluated using analysis of variances (ANOVA), and a chi-square test with post hoc comparisons using Sidak’s correction. The relationship between the depressive symptoms and diagnosis groups in regard to changes in HRQoL values was evaluated using a two-way analysis of covariance. Models included covariates for sex, age (biological), BMI, comorbidities (health and disease-related), education years and cohabiting (sociodemographic), when appropriate. A possible nonlinear relationship between BDI and adjusted change in HRQoL (15D) was assessed by using three-knot-restricted cubic spline regression models. Knot locations were based on Harrell’s recommended percentiles [26]. In the case of violation of the assumptions (e.g. non-normality) for continuous variables, a bootstrap-type method was used. The normality of variables was evaluated graphically and by using the Shapiro–Wilk W test. The Stata 17.0, StataCorp LP (College Station, TX, USA) statistical package was used for the analysis. The study protocol was approved by the Research Ethics Committee of the Northern Savo Hospital District (410/2016). ClinicalTrials.gov Identifier: NCT02992431. Registered 14/12/2016.

## Results

In the HA and DM groups there was almost the same number of patients and a bit over half were women. The CAD group was the smallest, and the group had a male majority. The ages in disease groups were different. The patients in the CAD group were the oldest and the patients in the DM group the youngest. Almost one in five DM patients had depressive symptoms, compared with only about one in eight people with HA. The HA patients weighed significantly less than the other two groups and the waists of both men and women were smaller. The DM patients had the highest burden of chronic diseases compared to the other two groups. The basic characteristics of the two study groups are presented in Table 1.

**Table 1.** Characteristics of the patients at baseline.

	Hypertension (H) *N* = 244	Coronary artery disease (C) *N* = 103	Diabetes (D) *N* = 240	*p*-value [multiple comparison]**
Women, *n* (%)	143 (59)	39 (38)	125 (52)	<0.001 [D/H,D/C]
Age, mean (SD)	70 (8)	72 (9)	67 (10)	0.002 [D/H, D/C]
Living with a spouse, *n* (%)	169 (69)	79 (77)	182 (76)	0.18
Number of educational years, mean (SD)	10.1 (2.9)	10.2 (3.1)	10.2 (3.2)	0.87
Retired, *n* (%)	215 (88)	94 (91)	208 (87)	0.48
Smoking, *n* (%)	23 (9)	6 (6)	32 (13)	0.092
Alcohol consumption, *n* (%)				0.50
Not at all	57 (24)	24 (24)	67 (28)	
One time per month or less	77 (32)	37 (36)	74 (31)	
2–4 times per month	74 (31)	25 (25)	68 (29)	
2–3 times per week	25 (10)	13 (13)	23 (10)	
4 or more times per week	9 (4)	3 (3)	6 (3)	
Physical activity *, mean (SD)	43 (18)	43 (20)	37 (21)	0.001 [D/H, D/C]
Blood pressure, mmHg, mean (SD)				
Systolic	148 (18)	144 (18)	144 (17)	0.027 [D/H]
Diastolic	84 (10)	81 (10)	80 (11)	<0.001 [C/H,D/H]
Body mass index, kg/m^2^, mean (SD)	27.9 (4.8)	28.2 (4.9)	31.3 (5.8)	<0.001 [D/H, D/C]
Waist, cm, mean (SD)				
Women	94 (14)	96 (15)	105 (15)	<0.001 [D/H, D/C]
Men	103 (11)	101 (11)	109 (13)	<0.001 [D/H, D/C]
HBA1C, mmol/mol, mean (SD)	37.3 (3.9)	38.4 (4.2)	46.0 (10.1)	<0.001 [D/H,D/C]
Fasting plasma glucose, mmol/l, mean (SD)	6.02 (0.57)	6.06 (0.78)	7.36 (1.50)	<0.001 [D/H,D/C]
LDL-C, mmol/l, mean (SD)	3.02 (0.96)	2.24 (0.79)	2.45 (0.90)	<0.001 [D/H,C/H]
Number of diseases, mean (SD)	1.8 (0.9)	2.6 (1.2)	3.1 (1.3)	<0.001 [C/H,D/H,D/C]
Beck depression index score, mean (SD)	5.4 (4.4)	5.7 (4.8)	6.6 (5.5)	0.005 [D/H]
Beck depression index score ≥10, *n*(%)	32 (13)	16 (16)	45 (19)	

*Kasari FIT index, % percentage, n number, SD standart deviation, HbA1c Haemoglobin A1c, LDL-C low-density lipoprotein cholesterol.

**Sidak’s multiple comparison procedure was used to correct significance levels for post hoc testing (*p* < 0.05).

As presented in Table 2, there were significant between-group differences in 15D dimensions at baseline in mobility, breathing, eating, usual activities, vitality and sexual activity, but only breathing and eating had significant between-group differences after adjusting for sex, age, BMI, comorbidities, education years and cohabiting.

**Table 2.** HRQoL was measured with 15D at baseline in hypertension (H), coronary artery disease (C) and diabetes (D) groups.

	Hypertension (H) *N* = 244	Coronary artery disease (C) *N* = 103	Diabetes (D) *N* = 240	*p*-values*	
	Mean (SD)	Mean (SD)	Mean (SD)	Crude	Adjusted**
Mobility	0.908 (0.145)	0.881 (0.163)	0.859 (0.190)	0.005 [D/H]	0.83
Vision	0.948 (0.109)	0.942 (0.111)	0.924 (0.146)	0.11	0.39
Hearing	0.912 (0.151)	0.895 (0.169)	0.905 (0.155)	0.64	0.79
Breathing	0.875 (0.186)	0.799 (0.217)	0.823 (0.213)	0.002 [C/H, D/H]	0.016 [D/C]
Sleeping	0.795 (0.192)	0.787 (0.180)	0.766 (0.215)	0.28	0.40
Eating	0.997 (0.032)	0.983 (0.077)	0.999 (0.023)	0.003 [D/H, D/C]	0.003 [C/H, D/C]
Speech	0.972 (0.087)	0.985 (0.064)	0.979 (0.076)	0.32	0.18
Excretion	0.834 (0.193)	0.817 (0.214)	0.835 (0.211)	0.73	0.30
Usual activities	0.901 (0.162)	0.837 (0.194)	0.862 (0.207)	0.006 [C/H]	0.12
Mental function	0.886 (0.177)	0.906 (0.163)	0.894 (0.168)	0.60	0.081
Discomfort and symptoms	0.704 (0.204)	0.695 (0.179)	0.685 (0.212)	0.61	0.093
Depression	0.922 (0.115)	0.908 (0.152)	0.897 (0.148)	0.14	0.85
Distress	0.907 (0.143)	0.885 (0.145)	0.894 (0.163)	0.39	0.34
Vitality	0.858 (0.146)	0.826 (0.138)	0.816 (0.175)	0.012 [D/H]	0.48
Sexual activity	0.864 (0.226)	0.760 (0.263)	0.778 (0.270)	<0.001 [C/H, D/H]	0.12
Total HRQoL	0.886 (0.083)	0.862 (0.096)	0.863 (0.102)	0.015 [D/H]	0.14

*Sidak’s multiple comparison procedure was used to correct significance levels for post hoc testing (*p* < 0.05).

**Adjusted for sex, age, BMI, comorbidities, education years and cohabiting.

The total change was not significant in the health-related quality of life, in HA patients (change 0.003 (95% CI: −0.004 to 0.011)), CAD patients (change −0.006 (95% CI: −0.017 to 0.005)) and DM patients (change 0.001 (95% CI: −0.007 to 0.009)) after adjusted for baseline, sex, age, BMI, comorbidities, education years and cohabiting.

**Figure 2.**
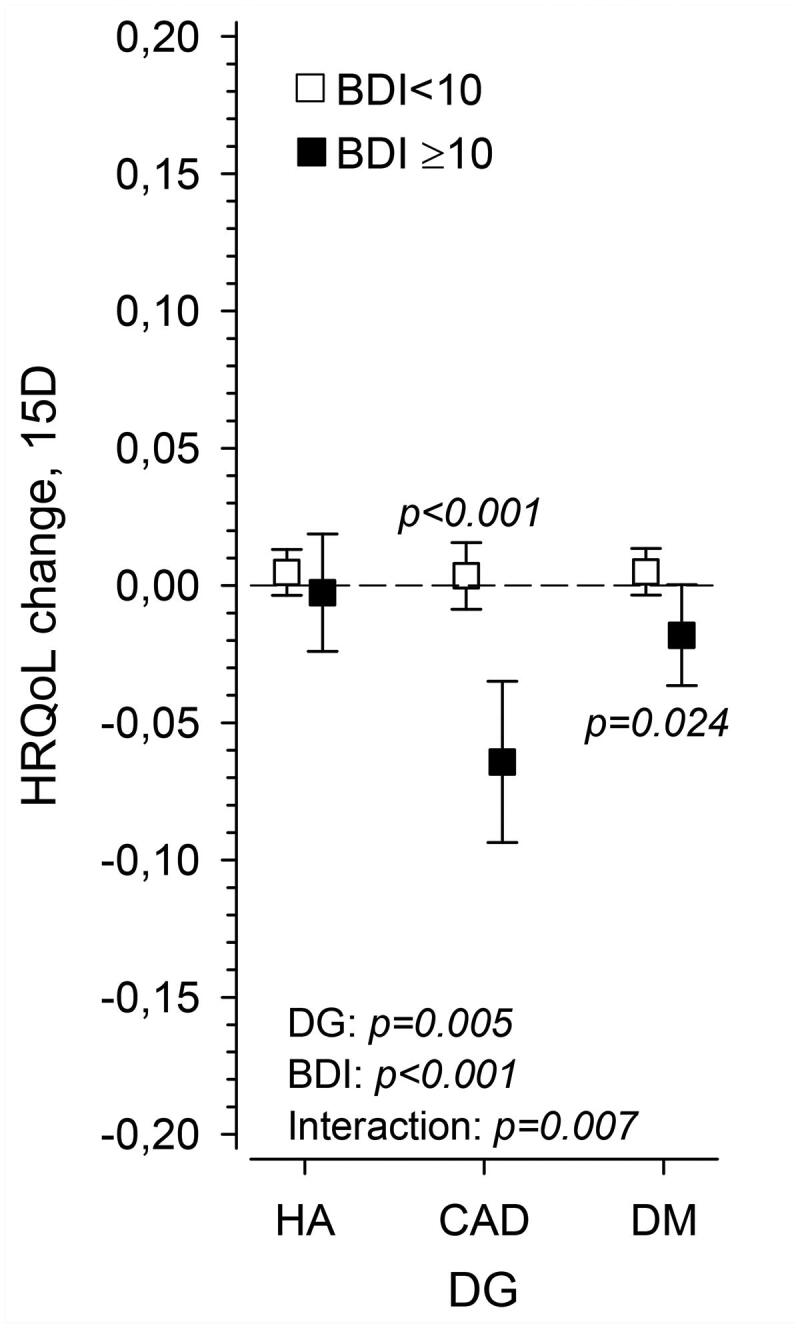
Change in HRQoL as measured with 15D in hypertension (HA), coronary artery disease (CAD) and diabetes (DM) groups in patients having a baseline BDI score ≥10 and <10. P-values adjusted for sex, age, baseline BMI, number of diseases, education years and cohabiting.

Figure 2 shows the HRQoL (15D) change in three disease groups separately for patients without (BDI <10) and with depressive symptoms (BDI ≥10). The change in 15D total score was significantly different between patients without and with depressive symptoms in CAD and DM patients. The P-value was adjusted for sex, age, baseline BMI, number of diseases, education years and cohabiting. In CAD patients the change was −0.064 (95% CI: −0.094 to −0.035) and in DM patients −0.018 (95% CI: −0.037 to 0.001).

**Figure 3.**
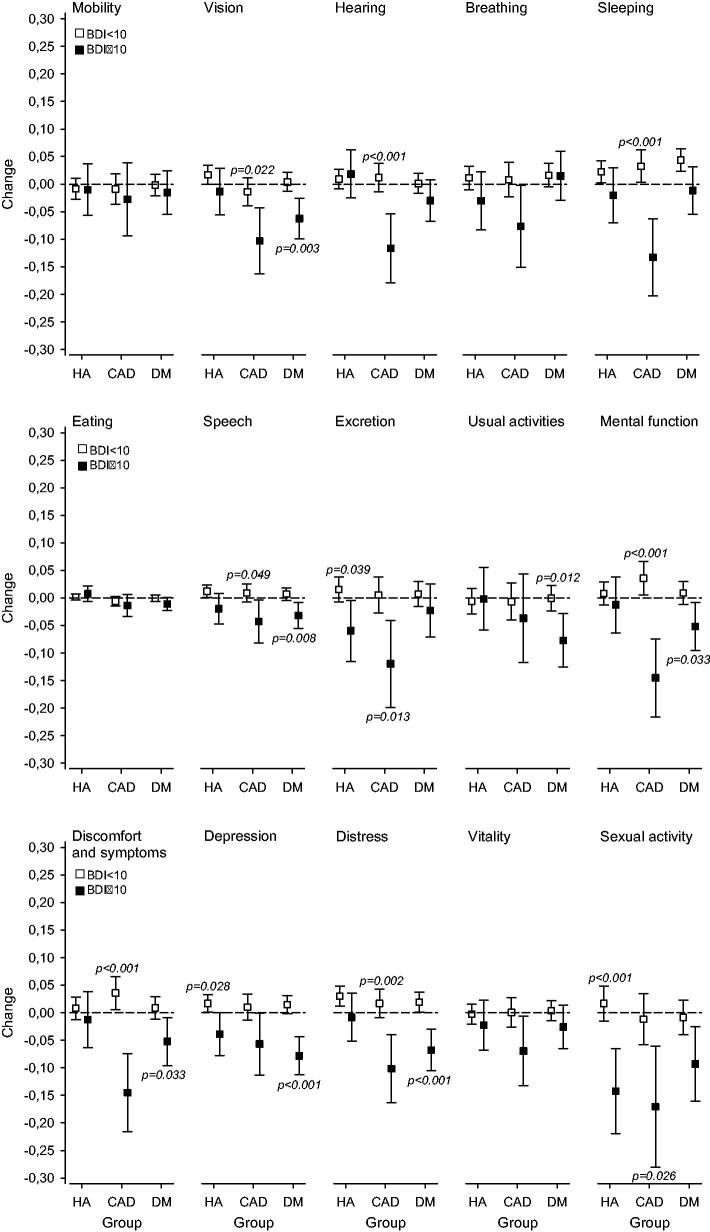
Presents the detailed changes in HRQoL measured with 15D according to BDI score at baseline adjusted for sex, age BMI, number of diseases, education years and cohabiting at the baseline in hypertension (HA), coronary artery disease (CAD) and diabetes (DM) groups.

Figure 3 shows us the changes in 15D dimensions after the 12-month follow-up according to the BDI score at baseline in three different disease groups adjusted for sex, age BMI, number of diseases, education years and cohabiting at the baseline.

In the HA group, there was a decrease in the 15D score related to depressive symptoms and a significant difference in change between patients without and with depressive symptoms in three dimensions (excretion, depression and sexual functions). In CAD patients the corresponding changes and differences were found in 9 dimensions (vision, hearing, sleeping, speech, excretion, mental functions, discomfort and symptoms, distress and sexual functions) and in DM patients in 7 dimensions (vision, speech, usual activities, mental function, discomfort and symptoms and distress).

**Figure 4.**
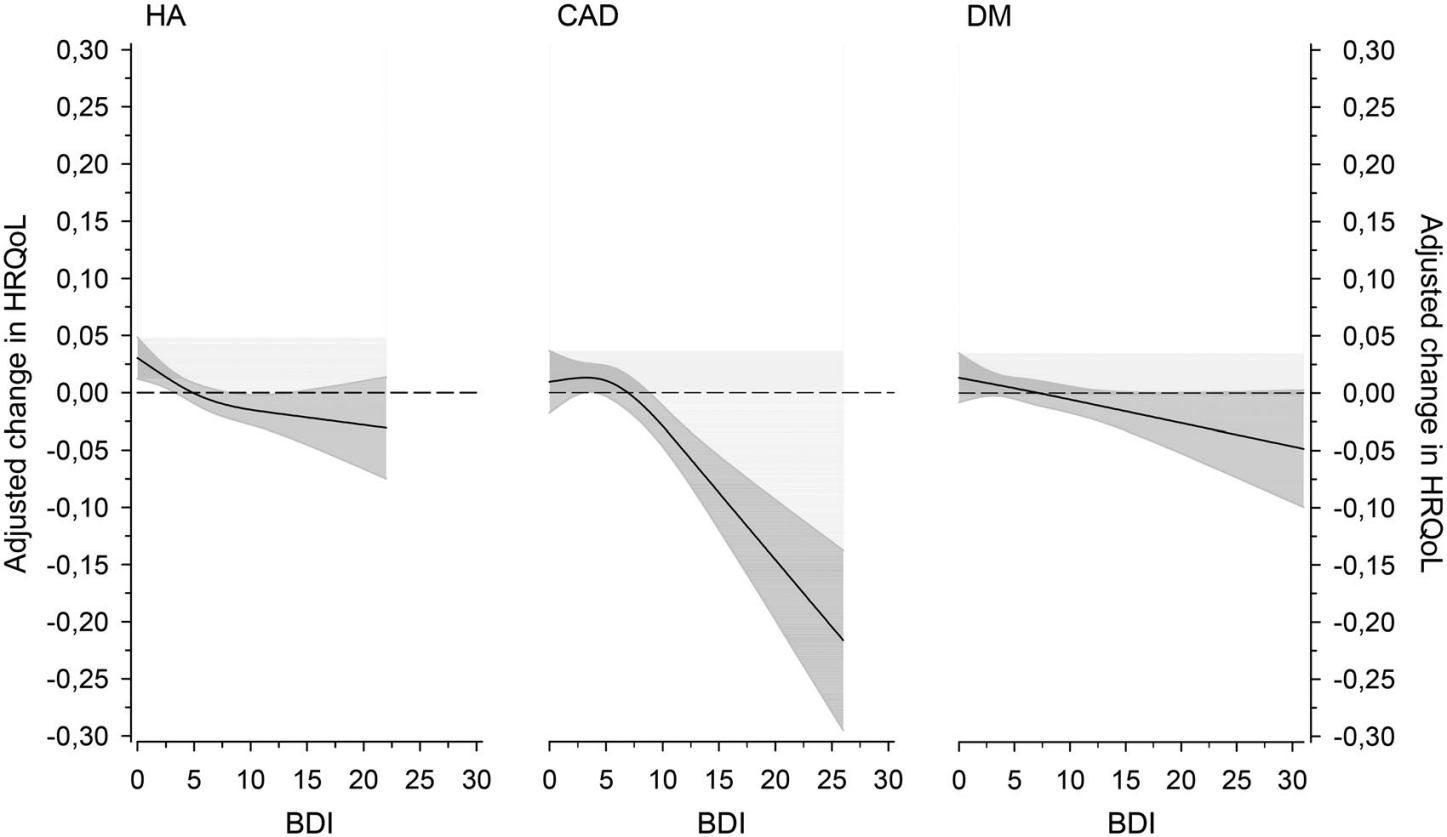
Relationships of adjusted change in HRQoL (15D) in three different disease groups (hypertension (HA), coronary artery disease (CAD) and diabetes (DM)) as a function of the BDI at baseline. The curves were derived from 3-knot-restricted cubic splines regression models. The models were adjusted for sex, age, baseline BMI, number of diseases, education years and cohabiting. The grey area shows the 95% confidence intervals.

Figure 4 shows the change in HRQoL after 12 months in three different disease groups (HA, CAD and DM) according to the BDI score at the baseline adjusted for sex, age, baseline BMI, number of diseases, education years and cohabiting. The level of HRQoL decreased significantly among patients with CAD and DM. There was a slight statistically non-significant decrease in the HRQoL of HA patients.

## Discussion

### Statement of principal findings

The main finding in our study was that baseline depressive symptoms were associated with a decrease in the health-related quality of life after 12 months of follow-up, particularly in patients with CAD and diabetes. To our best knowledge, this is the first study where a change in HRQoL was studied in relation to depressive symptoms in primary care patients with hypertension, CAD and diabetes in a 12-month follow-up setting. Interestingly, a significant decrease in HRQoL related to depressive symptoms was evident not only in mood-related dimensions but also in many other functions. The decrease was the widest in CAD patients, encompassing 9 dimensions, and the narrowest in hypertension with 3 dimensions.

A deterioration in HRQoL was associated with the severity of depressive symptoms at baseline in CAD and DM patients such that the greater the BDI score at the baseline, the greater the decrease in HRQoL. The change was not only statistically but also clinically significant in CAD and DM patients. Even mild baseline depressive symptoms seemed to predict worse HRQoL after a year.

### Strengths and weaknesses of the study

The strengths of this study include a 12-month follow-up in a primary healthcare environment with validated measures of HRQoL and depressive symptoms. The patients in our study were from the normal patient flow of the health centre, they were on average almost 70 years old with an average of more than two chronic diseases, representing patients of Finnish primary care. The number of patients was considerably high, and the dropout rate was low. The study was implemented in the everyday work in the health centre and consisted of patients with common chronic diseases treated in primary care. There are some limitations of this study that need to be acknowledged. We did not know the exact duration of the diseases although the patients were in regular follow-up due to their chronic diseases. In Finland, almost all adult patients followed up in primary care with diabetes have type 2 diabetes. Therefore, in the present study type of diabetes was not specified which can be regarded as a limitation. The study protocol did not include the diagnostic procedure to define definite psychiatric disorders. The reason to refuse to participate in the study was not known, so we did not know if the refused patients differed from those who participated and it is possible that the participants were more interested in taking care of themselves than patients not participating. Although the patients seemed to be quite representative of primary care patients, the study was conducted in one health centre, which means one must be cautious in generalizing these results.

### Findings in relation to other studies

Health-related quality of life is increasingly measured and reported as part of clinical trials and it is proven to be a relevant patient-related outcome in research and practice [7–11,27–35]. In the present study, we used the generic 15D measurement, which has been well-validated in Finland [19] and also in other countries [7,19,29,31,36].

The average HRQoL in Finland in a large population-based survey Health 2000 measured with 15D in the age group 65–74 years was 0.87 in both genders [6]. The mean HRQoL in our patients ranged from 0.862 to 0.886 at baseline. The HRQoL of HA patients in the present study was similar to the HRQoL found in hypertensive patients and quite close to the general population of the same age in an earlier Finnish population-based study [6]. In patients with HA, there was no deterioration of HRQoL during the follow-up indicating a stable situation of the disease. According to previous research, patients with HA and lower HRQoL have more symptoms, are aware of their disease, confront side effects from the medication [10,27] and have a higher BMI [10]. In our study, the patients were already aware of their disease and their medication had probably been stabilized over the years. The mean BMI of patients with HA was the lowest of the three studied disease groups in our study.

In the present study, patients with CAD had slightly higher HRQoL (0.862) compared with previous studies (0.777–0.859) [6,28–31] using 15D. In the previous follow-up studies with patients receiving optimal drug or operative treatment, the HRQoL increased [29–31]. In our study, the patients received regular treatment and follow-up in primary care, but we do not know if they had undergone some intervention due to their CAD during follow-up. Based on the low mortality (see Figure 1. flowchart), we can assume that patients had moderately stable disease. Previous studies have shown a decrease in HRQoL in cardiovascular disease patients if the disease is symptomatic or more severe [11,32], with depression, anxiety or hostility [2,11,32,37], higher BMI [28], age [11, 28], number of cardiovascular risks and female gender [11]. The increase in HRQoL has been demonstrated with effective treatment [30], higher patient education [11,32], marital status and social support [11]. The patients in our study had education for more than ten years on average and most of them were in a relationship.

The HRQoL of patients with diabetes was close to the findings from previous studies using 15D [6–8,33]. The previous research has indicated that the HRQoL in patients with DM is negatively influenced by a longer duration of DM [7,33,34], age [7,9,34], poor glycaemic control, low physical activity, presence of cardiovascular disease [9,34], high BMI [34,35], diabetes-related distress, chronic pain, mobility restrictions [35,38], comorbidities, low income [9] and low education level [9, 35]. Our DM patients with a mean age of 67 years had a chronic disease that was managed in the health centre for a long time. The patients with DM had the highest BMI and number of comorbidities among patients in our study. However, the mean HbA1C was quite low, indicating good glycemic control according to the recommendation of Finnish current care guideline [39]. Compared with CAD, the deterioration of HRQoL was less significant among patients with diabetes. Patients with diabetes were younger than patients with CAD, which may partly explain this finding. Another reason might be that CAD is probably perceived in a more dramatic way than diabetes with slower progress and good management.

There are a few studies on the association between HRQoL and depressive symptoms or depression related to CAD [2,4,16,37]. Two review articles have noted that HRQoL status changes in accordance with changes in depressive symptoms in CAD, and the history of depression is a significant predictor of HRQoL after hospitalization for acute coronary syndrome or rehabilitation after a coronary artery bypass grafting operation [2,37]. The depressive mood is a common reaction to an acute coronary event, but with comprehensively managed patients the depression can be transient [4]. Both depression and depressive symptoms in CAD patients increase morbidity and mortality [40–42]. Respectively, the association between lower HRQoL and depression has been found among patients with type 2 diabetes in a study based on a primary care setting [43]. Nevertheless, we lack research, especially from primary care on how depressive symptoms affect the health-related quality of life of patients with chronic diseases and their coping over time.

The findings of the present study indicate that even mild depressive symptoms may have a clinically important effect on future HRQoL. The prevalence of depressive symptoms in our study population was concordant with the previous studies [2,37,44–48]. The risk for increased depression among HA patients based on previous research is related to female gender, higher consumption of alcohol, smoking, higher BMI and low physical activity [48]. High BMI is a risk factor for both depression and low HRQoL in HA patients [10,48]. Weight, alcohol consumption, smoking and low physical activity are risk factors that we would be able to influence. In patients with CAD and DM, depression may reduce adherence to care, including self-care activity and the motivation to make lifestyle changes intended to reduce risks [2,44]. In patients with DM, depression is associated with an increased risk of mortality [49]. All this leads to more severe complications, lowering HRQoL [50] and possibly increasing depression. However, treating depression to remission and good continuity of care has been shown to manifest itself in better HRQoL [15,50].

### Meaning of the study

We suggest that our study provides new knowledge which makes it possible to find the patients whose HRQoL can be influenced. To be able to enhance the coping and prevent a decrease in the health-related quality of our patients with chronic diseases, it is important to respond to the needs of patients with coronary artery disease and diabetes having depressive symptoms. One aim for future research is to analyze methods to support patients even with mild depressive symptoms in order to maintain their HRQoL and adherence to care as well as possible.

## Data Availability

The datasets used and/or analysed during the current study are available from the corresponding author upon reasonable request.
